# Evaluation of SD Bioline HIV/syphilis Duo rapid test kits in Nepal

**DOI:** 10.1186/s12879-016-1694-9

**Published:** 2016-08-26

**Authors:** Geeta Shakya, Dipendra Raman Singh, Hemanta Chandra Ojha, Chet Raj Ojha, Shravan Kumar Mishra, Karishma Malla, Puspa Chaudhary, Kiran Regmi

**Affiliations:** 1 National Public Health Laboratory, Teku, Kathmandu, Nepal; 2 National Centre for AIDS and STD Control, Teku, Kathmandu, Nepal; 3 Paropakar Maternity Hospital, Kathmandu, Nepal; 4 Family Health Division, Teku, Kathmandu, Nepal

**Keywords:** Duo test, Sensitivity, Specificity, Kappa coefficient

## Abstract

**Background:**

Accurate and prompt diagnosis of HIV and syphilis simultaneously has reinforcing effect on their control program because of their prevalent co-infection. Availability of a simple user-friendly two-pronged and affordable detection tools brings down the cost of health care. They are important in the antenatal clinics, with added opportunity for intervention and prevention of mother to child transmission. In cooperation with rapid test kit manufacturers, SD Bioline, NPHL and NCASC, an evaluation of commercially available HIV/syphilis Duo rapid test kit (SD Bioline) to assess its performance and operational characteristics was done in the present study.

**Method:**

A prospective laboratory-based cross sectional study was conducted at a large Women’s Hospital. Ten thousand pregnant women, visiting the Hospital for antenatal care or for delivery, were enrolled in study. Tests were performed by the SD Bioline HIV/Syphilis Duo kit as well as national algorithm for HIV and syphilis diagnosis which were considered gold standard. Sensitivity, Specificity, positive predictive value and negative predictive value along with kappa coefficient were calculated for the kit under evaluation.

**Result:**

The sensitivity, specificity, Negative predictive value and Positive predictive value of the kit for HIV diagnosis were 100 % (95 % CI 83.18–100 %, 99.96–100 %, 83.18–100 %, and 99.96–100 %, respectively). Kappa value was found to be 1.0. Out of total cases, results of 9985 (99.85 %) cases were concordant with National algorithm for syphilis diagnosis. Thirteen (0.13 %) cases were found false positive while two were false negative. The sensitivity of the kit for syphilis diagnosis was found to be 95.45 % (95 % CI 84.86–98.74 %) and specificity was 99.87 % (95 % CI; 99.78–99.92 %). Positive predictive value was 76.36 % (95 % CI; 63.65–85.63 %) and Negative predictive value was 99.89 % (95 % CI; 99.39–99.99 %). Kappa value was found to be 0.85.

**Conclusion:**

The performance characteristics of SD Bioline HIV/Syphilis duo kit were found almost concordant with the kits being used for HIV and Syphilis diagnosis separately. Its implementation in antenatal clinics/VCTs could be an added opportunity for simultaneous diagnosis of HIV and syphilis.

## Background

Accurate and prompt diagnosis of HIV and syphilis is critical to disease control especially during the antenatal period with an additional opportunity for treatment and prevention of adverse effects of these disorders on the offspring.

Review of literature shows that co-infection with syphilis can increase the transmission of HIV by both increasing viral shedding through open ulcers [1, 2] and by increasing patient viral load [3, 4]. Simple user-friendly two-pronged and affordable detection tools could save lives and reduce overall costs borne by patients and health systems.

Rapid, accessible tests for simultaneous screening for HIV as well as syphilis are available on the market, but reliable information about their efficiency and dependability in quality or performance is coming up only gradually. Reliable evaluation reports of commercially available rapid diagnostic tests for tuberculosis [5] has been published but for syphilis and HIV large sized study reports of dual test kits are yet awaited.

Screening for HIV and syphilis is a basic routine test being an inseparable part of any antenatal check-up protocol and is also endorsed in the American Congress of Obstetricians and Gynecologists (ACOG) *Guidelines for Perinatal Care* [6]. Simultaneous screening for both these disorders would be cost-effective as well as time-saving. The current situation in Nepal entails the practice of testing separately for syphilis by screening by RPR test and confirmation by TPHA method and testing for HIV by following the *National algorithm for HIV rapid diagnosis* [7].

So, a rapid dual testing kit would meet the long felt need in the field as well as in the hospital settings. Hence, in cooperation with rapid test kit manufacturers, SD Bioline/NPHL, we undertook an evaluation of commercially available HIV/syphilis Duo rapid test kit (SD Bioline) to assess its performance, reproducibility and operational characteristics and to identify its utility as a reliable screening tool.

## Methods

The study was carried out with the cases enrolled at the antenatal clinic of Paropkar Maternity and Women’s Hospital (PMWH), Kathmandu, Nepal with the test-confirmation and backup-reference laboratory testing supported by the National Public Health Laboratory, Teku, Kathmandu, Nepal during the study period between July, 2014 and December, 2014.

Paropkar Maternity and Women’s Hospital is the largest maternity and gynecological hospital of the country serving population of the local embankment area communities as well as a large number of referral cases from all over the country. The National Public Health Laboratory is the apex laboratory institution of the country serving as a general and reference laboratory especially for investigations of public health importance.

All women attending the antenatal clinic of PMWH were enrolled in the study. This included all cases on their first visit to the ANC clinic as well as those on subsequent visits or for delivery, without being previously screened for HIV and/or syphilis. Women who were diagnostically confirmed cases or those already on therapeutic intervention with presumed diagnosis fell into the exclusion criteria. Informed written consent were taken in local language from all participants and ethical approval obtained from the ethical committee of the Nepal Health Research Council (NHRC).

Sample collection: Two types of samples were collected depending upon suitability for the tests and patient convenience: [1] blood collection by venipuncture for serum collection and [2] capillary blood collection by finger-prick method. All procedures for specimen collection were performed by laboratory personnels taking all biosafety precautions.

Test kit: SD BIOLINE HIV/syphilis Duo test kits (Standard Diagnostic Inc., Korea) were obtained for the evaluation study.

### Procedural steps (Fig. 1)

**Fig. 1:**
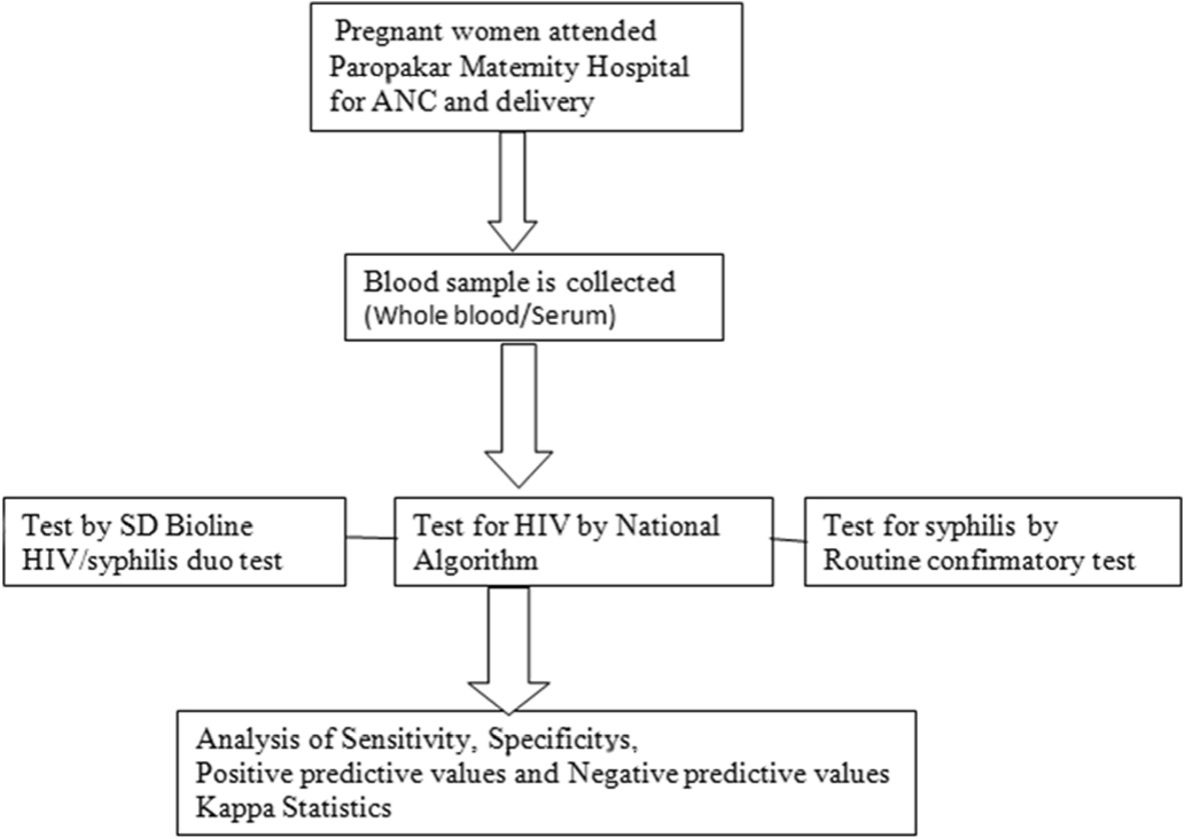
Procedural flow-chart

All the collected samples were tested using SD Bioline HIV/Syphilis duo test kit and after interpreting the test results as per manufacturer’s guideline entered into the records. In addition, all samples were simultaneously tested by using national HIV testing algorithm [7] with a combination of *Determine HIV-1/2/O assay (Abbott Laboratories, Abbott Park, IL), Uni-Gold Recombinant HIV-1/2 (Trinity Biotech, Bray, Ireland) HIV 1/2 Stat-Pak Ultra Fast (Chembio Diagnostic Systems, Medford, NY)*. Testing for syphilis was done, first by *RPR test by using IMMUTREP RPR test kit (Omega Diagnostics Scotland UK)* and all test positive samples were re-confirmed by TPHA method.

The used SD Bioline HIV/Syphilis duo test kits were preserved for dispatching to SD Bioline Company, South Korea.

Statistical analysis was carried out by using “openepi” online software (http://openepi.com/Diagnostic Test/DiagnosticTest.htm) for calculating sensitivity, specificity and predictive values. Kappa values was calculated according to the following formula:

$$ \mathrm{k}=\frac{{\mathrm{P}}^0\hbox{-} {\mathrm{P}}^{\mathrm{e}}}{1\hbox{-} {\mathrm{P}}^{\mathrm{e}}} $$

Where, P^0^ was observed agreement and P^e^ was expected agreement [8].

## Result

Total number of samples collected were 10,000, out of which 9,453 (94.53 %) were serum samples and 547 (5.47 %) were finger-prick whole blood samples as shown in Table 1. Of the women attending the ante-natal clinic 57.71 % were already in advanced pregnancy and near to delivery.

**Table 1:** Sample size and sample characteristics (*n* = 10,000)

Sample collection method:	Number of samples (%)
Venepuncture blood samples for serum collection	9,453 (94.53 %)
Finger-prick whole blood samples	547 (5.47 %)
Total number of samples collected by both methods	(9,453 + 547) = 10,000

**Table 2:** Assay results of the SD BIOLINE HIV/syphilis Duo test for dual detection of HIV and syphilis, Kathmandu, Nepal, 2014

RDT	Infection	RDT Result	Reference method^a^		
			Positive	Negative	Total
Using SD Bioline HIV/Syphilis duo test kit	Syphilis	Positive	42	13	55
		Negative	2	9943	9945
Total					10,000
	HIV	Positive	19	0	19
		Negative	0	9981	9981
Total					10,000

*RDT* rapid diagnostic test

^a^Tested by using national HIV testing algorithm [7] with a combination of *Determine HIV-1/2/O assay (Abbott Laboratories, Abbott Park, IL), Uni-Gold Recombinant HIV-1/2 (Trinity Biotech, Bray, Ireland) HIV 1/2 Stat-Pak Ultra Fast (Chembio Diagnostic Systems, Medford, NY)*. Testing for syphilis was done, first by *RPR test by using IMMUTREP RPR test kit (Omega Diagnostics Scotland UK)* and all test positive samples were re-confirmed by TPHA method. (Thirteen (0.13 %) cases in syphilis testing were found false positive while two were false negative and in case of HIV all of 19 cases tested positive using SD Bioline HIV/Syphilis duo test kit also tested positive by the reference method.)

### Testing for HIV

For HIV diagnosis, the sensitivity, specificity, negative predictive value and positive predictive value of the test kit were 100 % (95 % CI 83.18–100 %, 99.96–100 %, 83.18–100 %, and 99.96–100 %, respectively) and Kappa value was found to be 1.0. Information about the number of sample tested and result obtained by given methods are provided in Table 2.

#### Testing for syphilis

In the diagnosis of syphilis the test results of 9985 (99.85 %) samples using the test kit matched with that of the Reference method. Thirteen (0.13 %) cases were false positive and two were false negative. The sensitivity of the test kit for syphilis diagnosis was 95.45 % (95 % CI: 84.86–98.74 %) and specificity was 99.87 % (95 % CI: 99.78–99.92 %). Positive predictive value was 76.36 % (95 % CI; 63.65–85.63 %) and Negative predictive value was 99.89 % (95 % CI: 99.39–99.99 %) and Kappa value was found to be 0.85.

Of all samples tested for syphilis by the Reference method (RPR method) using IMMUTREP RPR test kits, 44 (0.44 %) tested positive and of those tested using SD Bioline HIV/Syphilis duo test kit 55 tests were positive. Only 13 (23 %) tests were positive for syphilis in the tests using HIV/Syphilis duo test kit and 2 (4 %) tested positive for syphilis using IMMUTREP RPR test kit in the whole series.

## Discussion

An evaluation of the diagnostic performance of the SD BIOLINE HIV/syphilis Duo test which is a two-pronged screening kit for simultaneous detection of HIV and syphilis, has been undertaken in the present study. The sensitivity and specificity of the kit in detecting HIV infection were found to be remarkably high at 100 %. These findings reiterate the results of earlier studies done in other countries [9, 10]. Comparable results have also been reported from the Southern Ethiopia, 2014 [11] study using the same test kit (shown in Table 3).

**Table 3:** Comparison of results of Southern Ethiopia and Kathmandu, Nepal studies

RDT	Southern Ethiopia, 2014^a^	Kathmandu, Nepal, 2014^b^
SD HIV/syphilis Duo	Infection	
% Sensitivity 95 % CI	HIV 100 (98.5 to 100)	100 % (95 % CI: 83.18–100 %)
	Syphilis 97.6 (92.4 to 99.6)	95.45 (95 % CI: 84.86–98.74 %)
% Specificity % CI	HIV 99.5 (97.6 to 100)	100 % (95 % CI: 99.96–100 %)
	Syphilis 96 (90.6 to 98.7)	99.87 (95 % CI: 99.78–99.92 %)
% PPV 95 % CI	HIV 99.5 (97.6 to 100)	100 % (95 % CI: 83.18–100 %)
	Syphilis 95.4 (89.3 to 98.5)	76.36 (95 % CI: 63.65–85.63 %)
% NPV 95 % CI	HIV 100 (98.5 to 100)	100 % (95 % CI: 99.96–100 %)
	Syphilis 98 (93.4 to 99.7)	99.89 (95 % CI; 99.39–99.99 %)

^a^Syphilis reference method, TPHA; HIV reference method, KHB/STAT-PAK/Unigold algorithm + ELISA. KHB, Shenghai Kehua Bioengineering; NPV, negative predictive value; PPV, positive predictive value; RDT, rapid diagnostic test. Ref: [11]

^b^Reference method: Tested by using national HIV testing algorithm [7] with a combination of *Determine HIV-1/2/O assay (Abbott Laboratories, Abbott Park, IL), Uni-Gold Recombinant HIV-1/2 (Trinity Biotech, Bray, Ireland) HIV 1/2 Stat-Pak Ultra Fast (Chembio Diagnostic Systems, Medford, NY)*. Testing for syphilis was done, first by *RPR test by using IMMUTREP RPR test kit (Omega Diagnostics Scotland UK)*

A rapid and sensitive tool for HIV testing has been a long felt need among health workers in the fields and the hospitals. Moreover, a two-pronged test kit designed for simultaneous HIV and syphilis detection has been a pressing need in the settings of the antenatal clinics and as a screening test for prospective mothers. SD BIOLINE HIV/syphilis Duo test has displayed many merits needed for such a test. The consequences of false negative test results in HIV testing of pregnant mothers have negative impact on efforts for the prevention of mother to child transmission of HIV infection (PMTCT). So, the results of the present evaluation as a reliable screening test for HIV without false negative test result need to be interpreted from these perspectives also. However, the SD BIOLINE HIV/syphilis Duo test’s usefulness for screening HIV-infected cases missed by other tests must be emphasized. Good screening tests do not replace the confirmatory reference tests but by their performances the consequences of false negative tests could be prevented to a great extent.

In the present study, for the diagnosis of syphilis The SD BIOLINE HIV/syphilis Duo test demonstrated a sensitivity of 95.45 % (95 % CI: 84.86–98.74 %) and specificity was 99.87 % (95 % CI; 99.78–99.92 %). The positive predictive value was 76.36 % (95 % CI; 63.65–85.63 %) and negative predictive value was 99.89 % (95 % CI; 99.39–99.99 %) and Kappa value was found to be 0.85. But these results have to be interpreted in the light of the fact that the predictive value of a test depends on the prevalence of a particular infection, and that the test may also demonstrate different performance elsewhere in lower prevalence regions. However, within the constraints of limited availability of diagnostic options in many resource-limited countries the SD BIOLINE HIV/syphilis Duo test can be a strategic tool for cost effective intervention in syphilis control through antenatal screening and treatment of positive cases [12].

Simple and easy method of dual screening of HIV and syphilis as observed in case of SD BIOLINE HIV/syphilis Duo test enhances the accessibility of quality screening service for most communities of the developing countries. Besides, this presents an opportunity to be automatically tested for syphilis for all those who get HIV testing without having to wait for the results of a second test. This may be an important step towards global elimination of HIV and syphilis in the pregnant women and other vulnerable risk groups.

Both HIV and syphilis being sexually transmitted diseases share common mode of transmission because of which simultaneous screening for both reinforces the control strategy of one by the other. By using this dual test kit as a point of care testing great impact can be expected in the control of both HIV and syphilis in the economically disadvantaged communities as point of care tests are strategic tools for the control and management of STIs [13].

## Conclusion

The performance characteristics of the SD Bioline HIV/Syphilis Duo test kit were found almost concordant with the reference kits currently being used for HIV and Syphilis diagnosis in Nepal. The performance of the kit was observed better for HIV diagnosis than for diagnosis of syphilis. Use of SD Bioline HIV/Syphilis Duo test in antenatal clinics/HTCs would meet the long felt need for a good screening tool for their day to day use with opportunity for simultaneous diagnosis of HIV and syphilis.

## Abbreviations

AIDS: Acquired immune deficiency syndrome
ART: Anti-retroviral therapy
DoHS: Department of health services
HIV: Human immunodeficiency virus
HP: Health post
HTC: HIV testing and counseling
NCASC: National center for AIDS and STD control
NPHL: National public health laboratory
PHC: Primary health care
PMTCT: Prevention of mother to child transmission
POCT: Point of care testing
RPR: Rapid plasma reagin
WHO: World health organization
